# Disparities by Race and Urbanicity in Online Health Care Facility Reviews

**DOI:** 10.1001/jamanetworkopen.2024.46890

**Published:** 2024-11-22

**Authors:** Neil K. R. Sehgal, Anish K. Agarwal, Lauren Southwick, Arthur P. Pelullo, Lyle Ungar, Raina M. Merchant, Sharath Chandra Guntuku

**Affiliations:** 1 Computer and Information Science Department, University of Pennsylvania, Philadelphia; 2 Leonard Davis Institute of Health Economics, University of Pennsylvania, Philadelphia; 3 Penn Medicine Center for Health Care Transformation and Innovation, University of Pennsylvania, Philadelphia; 4 Department of Emergency Medicine, University of Pennsylvania Perelman School of Medicine, Philadelphia

## Abstract

**Importance:**

Online review platforms offer valuable insights into patient satisfaction and the quality of health care services, capturing content and trends that traditional metrics might miss. The COVID-19 pandemic has disrupted health care services, influencing patient experiences.

**Objective:**

To examine health care facility numerical ratings and patient experience reported on an online platform by facility type and area demographic characteristics after the COVID-19 pandemic (ie, post-COVID).

**Design, Setting, and Participants:**

All reviews of US health care facilities posted on one online platform from January 1, 2014, to December 31, 2023, were obtained for this cross-sectional study. Analyses focused on facilities providing essential health benefits, which are service categories that health insurance plans must cover under the Affordable Care Act. Facility zip code tabulation area level demographic data were obtained from US census and rural-urban commuting area codes.

**Main Outcomes and Measures:**

The primary outcome was the change in the percentage of positive reviews (defined as reviews with ≥4 of 5 stars) before and post-COVID. Secondary outcomes included the association between positive ratings and facility demographic characteristics (race and ethnicity and urbanicity), and thematic analysis of review content using latent Dirichlet allocation.

**Results:**

A total of 1 445 706 reviews across 151 307 facilities were included. The percent of positive reviews decreased from 54.3% to 47.9% (*P* < .001) after March 2020. Rural areas, areas with a higher proportion of Black residents, and areas with a higher proportion of White residents experienced lower positive ratings post-COVID, while reviews in areas with a higher proportion of Hispanic residents were less negatively impacted (*P* < .001 for all comparisons). For example, logistic regression showed that rural areas had significantly lower odds of positive reviews post-COVID compared with urban areas (odds ratio, 0.77; 95% CI, 0.72-0.83). Latent Dirichlet allocation identified themes such as billing issues, poor customer service, and insurance handling that increased post-COVID among certain communities. For instance, areas with a higher proportion of Black residents and areas with a higher proportion of Hispanic residents reported increases in insurance and billing issues, while areas with a higher proportion of White residents reported increases in wait time among negative reviews.

**Conclusions and Relevance:**

This serial cross-sectional study observed a significant decrease in positive reviews for health care facilities post-COVID. These findings underscore a disparity in patient experience, particularly in rural areas and areas with the highest proportions of Black and White residents.

## Key Points

**Question:**

Is the COVID-19 pandemic associated with changes in online health care facility numerical ratings and patient experience across different facility types and area demographic characteristics?

**Findings:**

In this cross-sectional study of 1 445 706 online reviews from 151 307 US health care facilities between 2014 and 2023, the proportion of positive reviews decreased significantly, from 54.3% to 47.9%, after the COVID-19 pandemic. Rural areas, areas with a higher proportion of Black residents, and areas with a higher proportion of White residents experienced lower positive ratings; areas with a higher proportion of Hispanic residents were less negatively reviewed.

**Meaning:**

The findings of this study suggest that the COVID-19 pandemic is associated with a decrease in positive online reviews for health care facilities, revealing disparities in patient experience that vary by geographic and demographic factors.

## Teaser

This cross-sectional study examines changes in online platform reviews of health care facilities across the US from before to after the COVID-19 pandemic by area demographic characteristics.

## Introduction

Understanding patient experience is crucial for the health care sector, as it influences patient outcomes and the quality of care provided. Online review platforms (eg, Yelp and Google) offer valuable organically derived insights into patient experiences, capturing aspects that traditional surveys may miss.^1,2,3,4,5,6,7,8,9,10,11,12,13,14,15,16^ These platforms serve as a complementary barometer for patient expectations and satisfaction levels, reflecting the dynamics of health care service delivery.

Research of one online platform (Yelp) has observed that the reviews can supplement traditional surveys, providing nuanced insights into patient experiences across different health care settings.^1^ For instance, these reviews have covered a broader range of patient experience domains compared with traditional surveys, such as the Hospital Consumer Assessment of Healthcare Providers and Systems survey.^1^ Other studies have used online reviews to examine patient experiences of racism and discrimination in health care settings, revealing critical insights into these pervasive issues.^3,4^

The COVID-19 pandemic, with its major disruptions to health care services, provides a unique lens through which to examine these online reviews. The pandemic has brought about profound changes in health care, affecting service delivery and patient experiences worldwide. It also exposed and exacerbated racial and ethnic disparities in health care access and outcomes. As the health care sector navigates the aftermath of the pandemic, maintaining and improving patient trust and care quality have become paramount. However, less is known about the extent and nature of changes in online health care reviews in response to the COVID-19 pandemic across various types of health care facilities and demographic groups. Understanding these changes is crucial for informing targeted interventions and quality improvement efforts, particularly in underserved communities. Moreover, insights from online reviews may guide health care professionals and policymakers in addressing gaps in service delivery and enhancing patient-centered care during and after public health crises. This work is essential to help adapt health care practices to evolving patient needs and ensure equitable care across different demographic and geographic settings.

This study sought to explore quantitatively and qualitatively how online reviews of health care facilities have evolved before and after the onset of the COVID-19 pandemic (post-COVID). By using a comprehensive dataset of all health facility reviews from the platform studied (Yelp) over a 10-year period between January 1, 2014, and December 31, 2023, this study aimed to investigate 3 questions: (1) Did the health facility reviews change numerically before and after March 2020? (2) Did reviews change numerically across facility type and demographic characteristics, namely, race and urbanicity? and (3) Did the patient experience change, as measured by topic modeling analyses, before and after March 2020?

## Methods

This cross-sectional study was deemed exempt from review by the University of Pennsylvania Institutional Review Board because it did not meet the definition of human participant research. The Strengthening the Reporting of Observational Studies in Epidemiology (STROBE) reporting guideline was followed.

### Data Collection

The data used in this study came from an academic dataset generated directly by the platform for research. The dataset includes all US facilities tagged as health and medical according to the platform’s developer documentation. The dataset is updated daily and includes information not available through the application programming interface. This study collected all health and medical facility reviews between January 1, 2014, and December 31, 2023. Each review contained information on the facility category and subcategory, facility zip code, date of review, numerical rating, and review text. The health and medical category includes many health subcategories and can potentially introduce bias if analyzed in aggregate. We filtered the data to facility subcategories that could be considered essential health benefits, services that the Affordable Care Act mandates health insurance plans cover.^17^ The facility subcategories were additionally reviewed and verified by one of us (A.K.A.) with experience in this research area to ensure completeness.^7,11,13^ The facilities included were urgent care clinics, medical centers, physical therapy, pharmacies, physicians, hospitals, counseling and mental health, rehabilitation centers, emergency departments, occupational therapy, home health care, skilled nursing, ultrasonography imaging centers, lactation services, prenatal care, speech therapists, diagnostic services, reproductive health centers, nurse practitioners, and dialysis clinics.^5^ Facility types not deemed essential and excluded included acupuncture, body contouring, cannabis clinics, cryotherapy, herbal shops, and oxygen bars. A review’s facility field could list multiple subcategories (eg, both hospital and emergency department). The reviews are scored from 1 to 5, with most scores following a bimodal distribution of 1 or 5 (eFigure 1 in Supplement 1). For analysis purposes, we created a positive review indicator variable for reviews with 4 or more stars (of a maximum of 5 stars).

### Variables

Previous research has highlighted the role of neighborhood demographic characteristics in shaping health care access and disparities, which can be reflected in patient reviews and satisfaction.^18^ To gather proxy demographic information on the areas surrounding each health care facility, we mapped each facility’s zip code to the corresponding zip code tabulation area (ZCTA) using a crosswalk.^19^ We collected data on the median income, which is used as a potential covariate in the analyses, and the percentage of Black, Hispanic, and White populations from census data for each ZCTA.^20^ These variables were categorized into quartiles for analysis. The percentages for Black and White populations are not mutually exclusive, eg, a facility could be in a ZCTA that simultaneously falls into the highest quartile for both the percentage Black and percentage White populations. For example, a ZCTA could be 13% Black and 81% White and be in the highest quartile for both the percentage Black and percentage White categories. Additionally, we applied rural-urban commuting area codes to determine whether each facility was located in an urban or rural setting, assigning an indicator variable to each facility based on these codes.^21^ To assess for changes after the onset of the COVID-19 pandemic, we created an indicator variable for reviews written after March 31, 2020, the month following the World Health Organization’s pandemic declaration.^22^

### Review Content and Facility Demographics

We leveraged the individual advantages of both latent Dirichlet allocation (LDA), a machine learning approach that identifies co-occurring words and groups them into topics, and nonnegative matrix factorization (NMF) to analyze and refine themes from reviews.^23^ Initially, LDA was applied to generate a broad array of topics.^24^ This was achieved using the differential language analysis toolkit mallet interface.^25^ Topic coherence and uniqueness measures were used to determine the preliminary number of topics.^26,27^ For robustness, we additionally experimented with BERTopic, but this method yielded poor coherence scores and was not used in the final analysis.^28^ Subsequently, NMF was used to further distill our LDA-generated topics into a more concise set.^29^ The refined topics were then categorized into themes by manual review. To assess changes in themes over time, we compared the mean prevalence of each theme before and after March 2020. To assess changes over time across demographic variables, we ran a linear regression of the prevalence of each theme on an interaction between demographic variables with a post-COVID indicator and a fixed effect for the ZCTA. We ran these regressions on subsets of the data limited to only the reviews of 4 stars or more and less than 4 stars to understand how positive and negative reviews changed separately.

### Statistical Analysis

Two-sided, unpaired *t* tests were used to test for significant changes in review positivity before and post-COVID by review characteristics. Logistic regression models were used to assess the association between positive ratings and facility characteristics. Logistic regressions with a ZCTA fixed effect and interactions between demographic variables and a post-COVID indicator were used to test whether certain regions saw large decreases in positive reviews after March 2020. Multivariate regression was used to assess the independent associations between facility type, region demographic variables, and time. As a robustness check, we conducted an interrupted time series analysis to assess changes in the slope and level of positive reviews before and after the onset of the COVID-19 pandemic, with separate analyses for facilities in the top and bottom quartiles of racial and ethnic composition and urban and rural settings. Interrupted time series was performed on monthly aggregated data overall and quarterly aggregated data for specific facility types and ZCTA characteristics to account for the high variability from the modest sample size across some months. All statistical analyses were conducted in Python, version 3.9.6 (Python Software Foundation). The significance threshold was *P* < .05.

## Results

A total of 6 450 146 reviews across 601 252 facilities were collected. Of these, we excluded 5 004 440 reviews from 449 945 facilities belonging to health categories deemed nonessential according to the Affordable Care Act.^17^ The final dataset consisted of 1 445 706 reviews across 151 307 facilities. The median number of reviews per facility was 3 (IQR, 6) with high heterogeneity by facility type (eTable 1 in Supplement 1). Most of the facilities (93.3%) were urban. The median racial and ethnic demographic characteristics of these facilities were as follows: percentage Black, 5 (IQR, 2-12); percentage Hispanic, 13 (IQR, 7-25), and percentage White, 68 (IQR, 52-80). Summary statistics of facility types are displayed in the Table.

**Table.** Summary Statistics of Online Reviews and Health Care Facilities

Type of facility	No. (%)	
	Reviews (n = 1 445 706)	Facilities (n = 151 307)
Urgent care	292 916 (20.3)	11 535 (7.6)
Medical center	278 924 (19.3)	18 961 (12.5)
Physical therapy	223 894 (15.5)	28 246 (18.7)
Pharmacy	197 095 (13.6)	17 248 (11.4)
Hospital	171 895 (11.9)	8436 (5.6)
Physician	106 268 (7.4)	24 501 (16)
Counseling and mental health	95 623 (6.6)	21 418 (14)
Rehabilitation center	63 385 (4.4)	8939 (5.9)
Emergency department	46 668 (3.2)	1220 (0.8)
Occupational therapy	45 536 (3.1)	6318 (4.2)
Home health care	43 270 (3.0)	9669 (6.4)
Skilled nursing	31 287 (2.2)	4051 (2.7)
Ultrasonography imaging center	30 359 (2.1)	1349 (0.9)
Lactation service	20 663 (1.4)	1444 (1.0)
Prenatal	20 584 (1.4)	1328 (0.9)
Speech therapist	17 999 (1.2)	3223 (2.1)
Diagnostic service	15 766 (1.1)	1335 (0.9)
Reproductive health service	14 775 (1.0)	719 (0.5)
Nurse practitioner	7005 (0.5)	1296 (0.9)
Dialysis clinic	1436 (<0.1)	750 (0.5)

**Figure 1.**
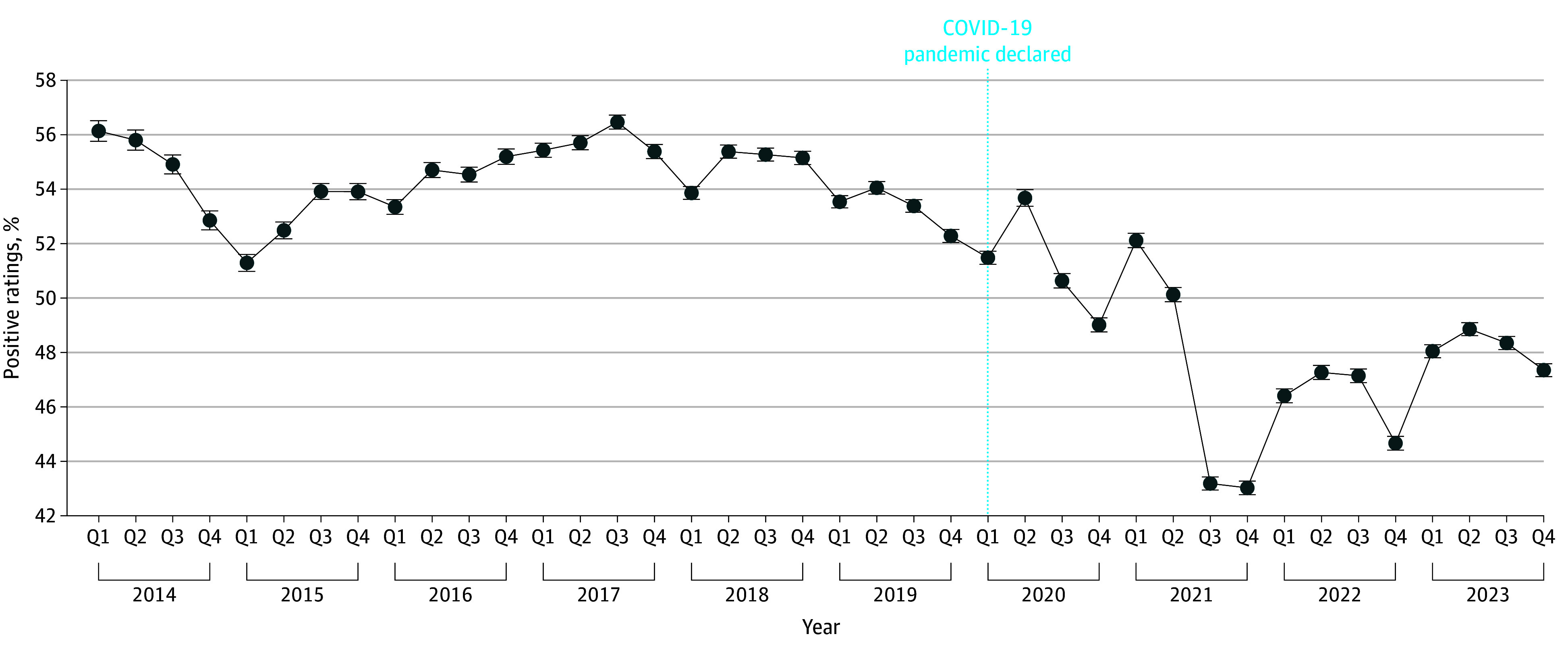
Percent of Online Platform Health Facility Ratings of 4 or Greater by Year Error bars indicate 95% CI.

We found that average reviews post-COVID (March 31, 2020, to December 31, 2023) decreased in positivity by 6.38 percentage points (from 54.3% to 47.9%; *P* < .001), with steep decreases seen beginning in the second quarter of 2021 (Figure 1). In general, we found that this decrease held across facilities (eFigure 2 in Supplement 1). There were a few noticeable exceptions. For instance, reproductive health service review positivity increased by 5.0 percentage points (*P* < .001) after March 2020.

**Figure 2.**
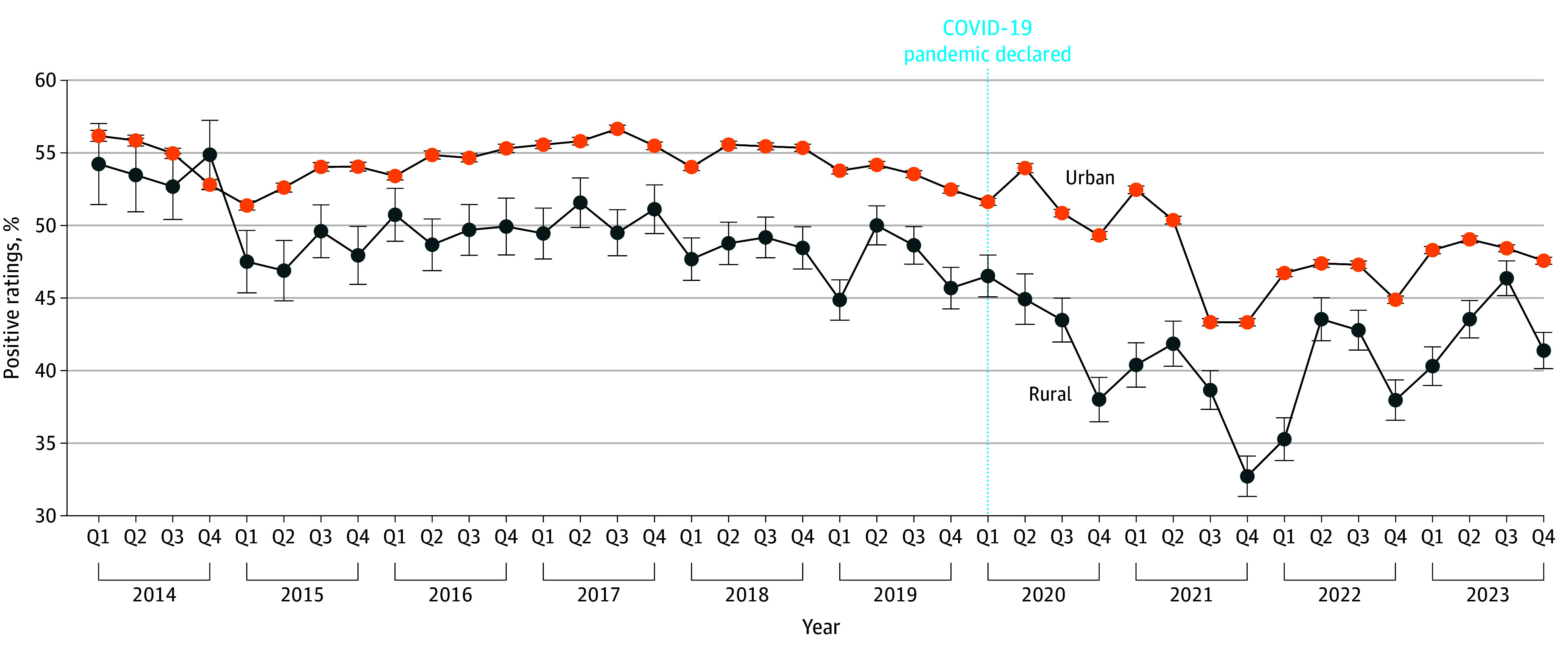
Percent of Online Platform Health Facility Ratings by Urban or Rural Setting Over Time Error bars indicate 95% CI.

We found that rural areas had fewer positive reviews (odds ratio [OR], 0.77; 95% CI, 0.72-0.83), and a regression with an interaction revealed these areas witnessed larger decreases relative to urban areas after March 2020 (OR, 0.93; 95% CI, 0.87-0.98) (Figure 2). The results were robust when controlled for median income.

**Figure 3.**
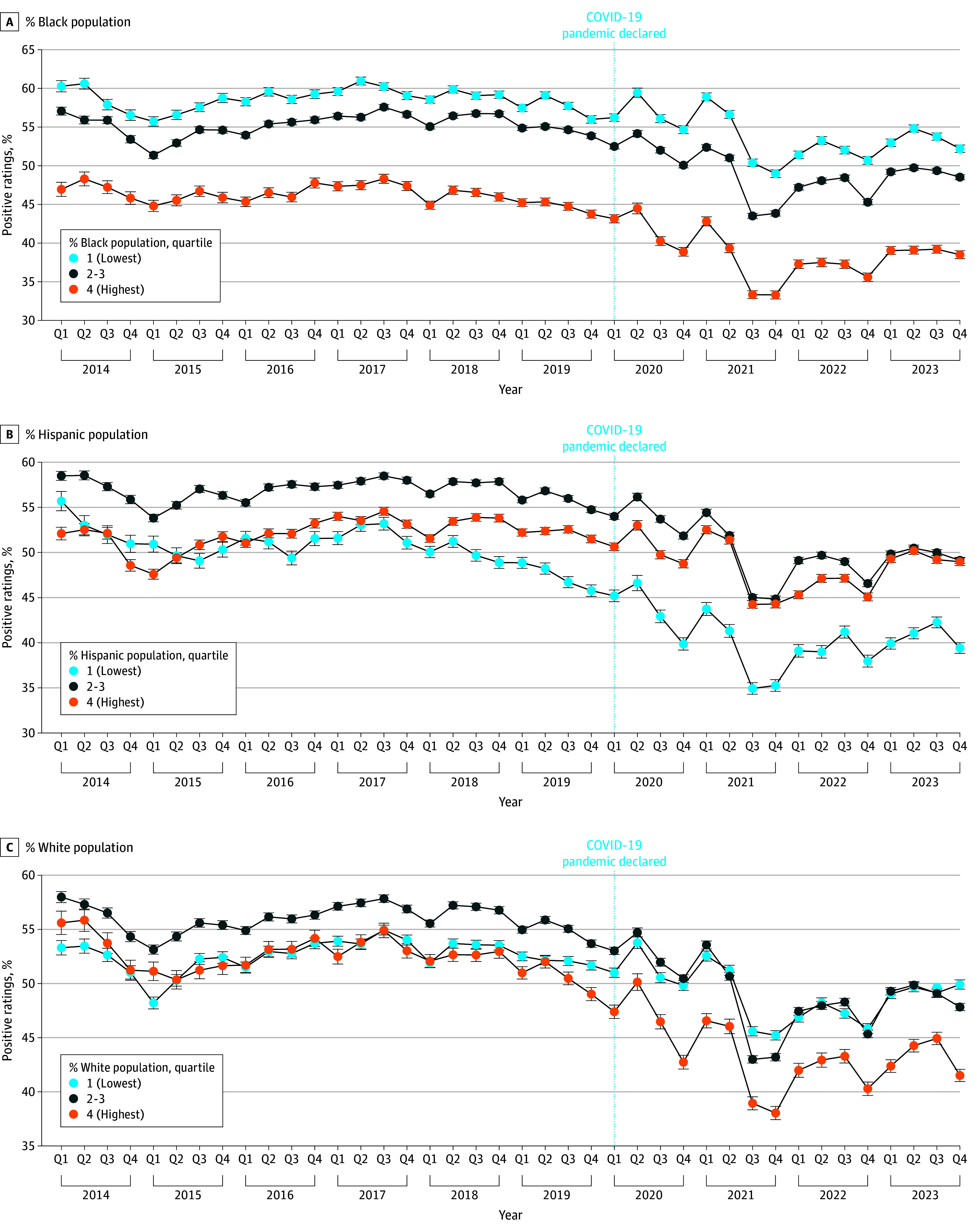
Percent of Online Platform Health Facility Ratings by Race and Ethnicity Quartile Over Time Error bars indicate 95% CI.

Areas with the highest proportions of Black residents had worse reviews pre-COVID and had larger decreases in positive reviews post-COVID. Areas with the highest proportions of White residents also saw the largest decreases in positive reviews post-COVID. However, areas with the highest proportion of Hispanic residents had better reviews pre-COVID and had smaller decreases in positive reviews post-COVID (*P* < .001 for all comparisons) (Figure 3). These results were robust (ie, all findings were statistically significant) when controlled for median income. Interrupted time series analysis suggested that the onset of the COVID-19 pandemic did not result in an immediate decrease in review positivity levels, but there was a significant decrease in review positivity over time, approximately equivalent to 1% every 10 months (eFigure 3 in Supplement 1). Additionally, there was substantial heterogeneity in these changes across facility types and ZCTA characteristics (eTable 2 in Supplement 1).

Multivariate analyses found that the pre-COVID or post-COVID time period, specific facility types, urban and rural location, and race and ethnicity are independently associated with review ratings (all *P* < .001) (eTable 3 in Supplement 1). The OR for positive reviews before and after March 2020 was 0.73 (95% CI, 0.66-0.81). Facility types with lower rates of positive reviews included rural facilities, facilities located in areas with higher proportions of Black residents, and facilities located in areas with higher proportions of White residents. Results were generally robust when controlled for median income; however, rural facilities were associated with higher rates of positive reviews.

### Patient Experience

**Figure 4.**
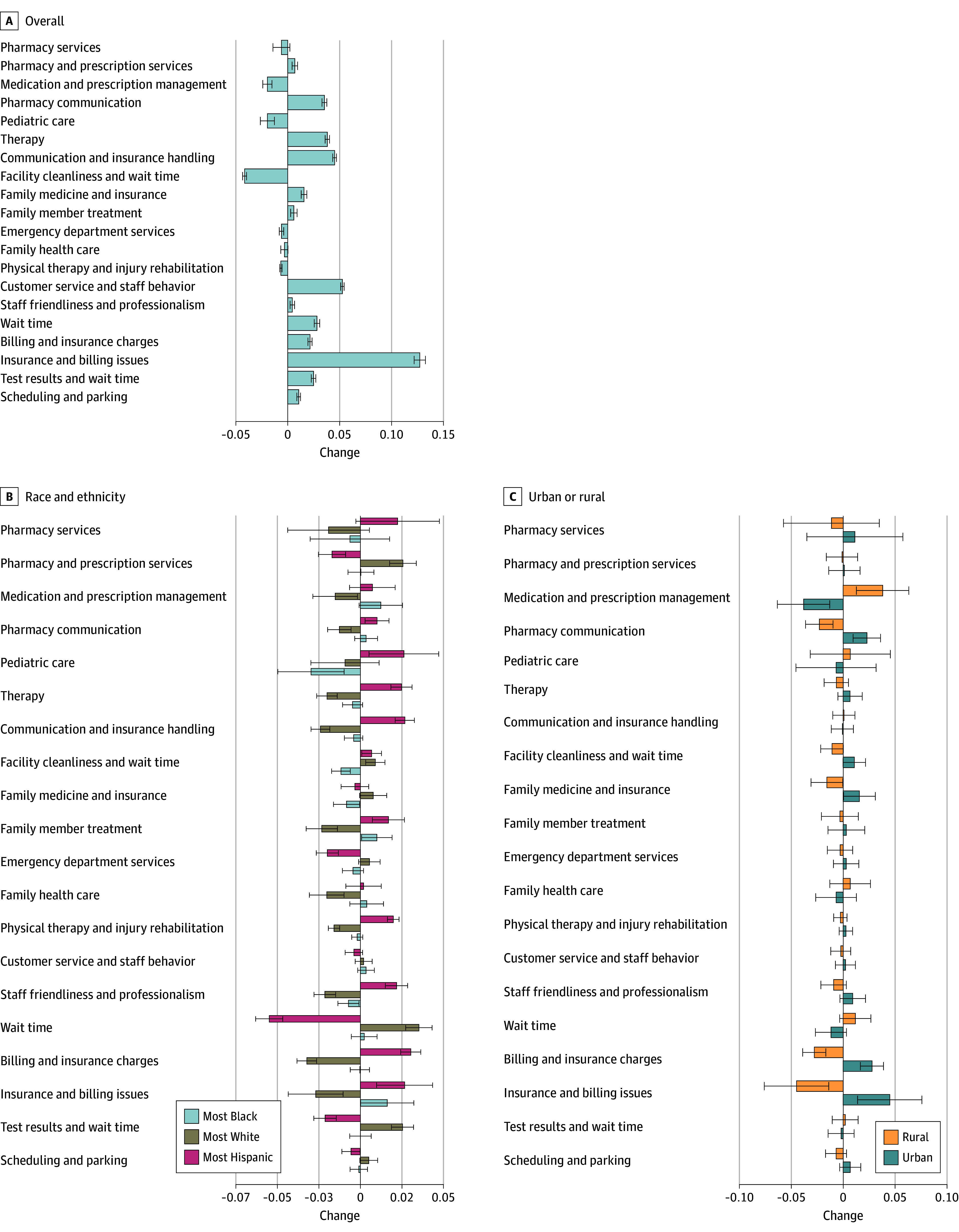
Change in Theme Prevalence Over Time Among Negative Reviews Error bars indicate 95% CI.

Latent Dirichlet allocation with 200 topics achieved the best balance of coherence and uniqueness (eTable 4 in Supplement 1), with NMF used to distill these 200 topics down to 20 themes. eTable 5 in Supplement 1 lists the 20 themes identified across all reviews along with the top 10 words for each theme. Examples of themes include scheduling and parking, billing and insurance charges, communication and insurance handling, and waiting time. Figure 4 displays the prevalence of these themes over time among negative reviews. eFigure 4 in Supplement 1 shows the results for positive reviews.

Figure 4 presents coefficients from a regression of the theme prevalence on the post-March 2020 indicator and percentage race and ethnicity quartiles. For example, among negative reviews, reviews were more likely to mention insurance and billing issues in the highest quartile of areas with Black residents and highest quartile of areas with Hispanic residents compared with the lowest quartile of Black and lowest quartile of Hispanic residents after March 2020. In addition, the areas with the highest quartile of White residents were more likely compared with the lowest quartile White areas to mention wait time among negative reviews post-COVID.

Figure 4 presents coefficients from a regression of the theme prevalence on the post-March 2020 indicator and an urban or rural indicator variable. Among negative reviews, the medication and prescription management theme increased significantly for rural areas compared with urban areas after March 2020. In addition, the insurance and billing issues theme decreased in rural areas compared with urban areas after March 2020.

## Discussion

This study aimed to examine changes in online platform reviews of health care facilities over time, within the context of the pandemic and across demographic variables. We found that, in general, ratings decreased significantly post-COVID. Rural areas, areas with the highest proportion of Black residents, and areas with the highest proportion of White residents saw the largest decreases in positive reviews; however, there was significant heterogeneity by facility type. Areas with the higher proportions of Hispanic residents had smaller decreases in positive reviews post-COVID, which may be due to differing patterns of use of the platform among ethnic groups or the relative stability of facilities in these areas, although further research is needed to explore this finding.

Topic modeling results revealed distinct issues facing each community. For instance, among negative reviews, insurance and billing issues increased in prevalence post-COVID, with the areas with the highest proportion of Black and Hispanic residents seeing significant increases. The areas with the highest proportion of White residents were more likely to mention wait time among negative reviews post-COVID. Rural areas were more likely to mention medication and prescription management post-COVID compared with urban areas among negative reviews.

Our findings align with and expand on previous research highlighting changes in patient experiences during the COVID-19 pandemic. Prior studies have reported that the pandemic introduced widespread challenges for health care delivery. For example, one study of the Hospital Consumer Assessment of Healthcare Providers and Systems survey found large decreases in satisfaction with hospitals in 2021.^30^ The observed decrease in positive reviews across many facility types aligns with these findings, reinforcing the notion that the pandemic disrupted normal health care operations and patient expectations. Moreover, the increased prevalence of topics related to communication issues and billing challenges aligns with earlier studies indicating that these are critical areas affecting patient satisfaction.^31,32^ The identified changes also complement earlier work suggesting that certain demographic groups, such as rural communities and predominantly Black areas, face unique health care challenges exacerbated by the COVID-19 pandemic.^33,34,35^ Additionally, our study suggests the utility of online platform reviews as a valuable data source for health systems and policymakers to monitor patient sentiment and identify area-specific issues. By leveraging this real-time, granular feedback, health care systems and policymakers can develop targeted interventions that address the specific concerns of different communities, thereby improving overall patient satisfaction and health care delivery.

These findings have important implications for health care professionals and policymakers. The reduction in positive reviews across many facilities post-COVID suggests a need for increased attention to patient experience, especially during public health emergencies. The heterogeneity in review patterns across facility types and demographic variables indicates that targeted interventions may be needed to address specific issues faced by different communities and health care services. For example, the increased negative mentions of insurance and billing issues in areas with a higher proportion of Black residents post-COVID suggest additional attention and support for improved billing infrastructure in these communities. The prominence of billing and communication issues in post-COVID reviews underscores the importance of administrative efficiency and patient communication in maintaining patient satisfaction.

### Limitations

Several limitations must be considered in interpreting the study results. First, our study examined patient reviews on only 1 online review platform and the results may not be generalized to other platforms. Additionally, users of this online review platform may not be representative of the general population. Moreover, online reviews can inherently overrepresent dissatisfied customers, especially during public health crises such as the COVID-19 pandemic, when heightened frustration may lead to more negative reviews. Similarly, the possibility of decreased review activity due to various pandemic-related factors (eg, reduced health care use) could affect the findings. It is also possible that the platform changed its algorithms for filtering and displaying reviews, as well as its interface, over the 10-year study period, which could have influenced user behavior, review visibility, and the overall patterns of reviews. Future studies should consider such platform-related dynamics when interpreting results from online review data. Second, while our analysis incorporated neighborhood-level demographic factors to understand community influences on patient reviews, we acknowledge the risks of ecological fallacy in this approach. Many health care facilities serve diverse populations that extend beyond their immediate ZCTAs, and as such, neighborhood characteristics may not fully capture the diversity of patients’ experiences. Future research could benefit from more granular data that capture patient demographic factors directly or consider additional facility-level characteristics that more accurately reflect patient interactions and satisfaction. Third, we restricted the study to health care facilities that we deem essential health benefits, and the results may differ for other categories. In addition, certain categories, such as pharmacy, may include reviews from non–health-related activities of the facility.

In addition to the impact of the COVID-19 pandemic, other major events and policy changes during the study period, such as the CARES Act, likely influenced review patterns. The CARES Act provided financial support to health care facilities that may have helped stabilize operations and maintain patient satisfaction despite pandemic-related challenges. Furthermore, the rapid adoption of telehealth, driven by the need for safer health care delivery options, could have shaped patient perceptions of accessibility and convenience, affecting both positive and negative review changes. Future research could further explore how these policy shifts and the telehealth expansion specifically contributed to changes in online reviews.

## Conclusions

Our cross-sectional analysis highlights substantial shifts in online reviews of health care facilities post-COVID, with important variations across facility types and demographic regions. The findings underscore the need for tailored interventions to improve patient experience, address systemic issues in health care environments, and respond to the changing expectations and needs of health care consumers. Future research should further explore the factors associated with these issues and evaluate interventions to enhance patient satisfaction and mitigate negative influences on patient experience.

## Notes

Supplement 1. **eFigure 1.** Distribution of Platform Health Facility Ratings, 2014-2023 **eTable 1.** Median Number of Reviews by Facility Type **eFigure 2.** Difference in Mean Platform Health Facility Positive Rating Pre/Post March 2020 **eFigure 3.** Interrupted Time Series (ITS) Analysis of Positive Reviews Pre- and Post-COVID-19 **eTable 2.** Interrupted Time Series (ITS) Analysis of Positive Reviews Pre- and Post-COVID-19: Coefficients and 95% Confidence Intervals for Changes in Review Positivity by Facility Type, Racial/Ethnic Composition, and Urban/Rural Settings **eTable 3.** Multivariate Relationship Between Platform Health Facility Rating Positivity and Facility Characteristics and Time **eTable 4.** Topic Coherence and Uniqueness Scores by Model **eTable 5.** Themes Associated With Platform Health Facility Reviews **eFigure 4.** Change in Theme Prevalence Over Time Among Positive Reviews

Supplement 2.
Data Sharing Statement
