# Supplementary material for: Disparities by Race and Urbanicity in Online Health Care Facility Reviews
Source: JAMA Netw Open. 2024 Nov 22;7(11):e2446890. doi: 10.1001/jamanetworkopen.2024.46890 (PMC11584935; doi:10.1001/jamanetworkopen.2024.46890)
Supplement: Supplement 1. — eFigure 1. Distribution of Platform Health Facility Ratings, 2014-2023 eTable 1. Median Number of Reviews by Facility Type eFigure 2. Difference in Mean Platform Health Facility Positive Rating Pre/Post March 2020 eFigure 3. Interrupted Time Series (ITS) Analysis of Positive Reviews Pre- and Post-COVID-19 eTable 2. Interrupted Time Series (ITS) Analysis of Positive Reviews Pre- and Post-COVID-19: Coefficients and 95% Confidence Intervals for Changes in Review Positivity by Facility Type, Racial/Ethnic Composition, and Urban/Rural Settings eTable 3. Multivariate Relationship Between Platform Health Facility Rating Positivity and Facility Characteristics and Time eTable 4. Topic Coherence and Uniqueness Scores by Model eTable 5. Themes Associated With Platform Health Facility Reviews eFigure 4. Change in Theme Prevalence Over Time Among Positive Reviews [file jamanetwopen-e2446890-s001.pdf]

Supplementary Online Content

Sehgal NKR, Agarwal AK, Southwick L, et al. Disparities by race and urbanicity in online health care facility reviews. *JAMA Netw Open*. 2024;7(11):e2446890. doi:10.1001/jamanetworkopen.2024.46890

eFigure 1. Distribution of Platform Health Facility Ratings, 2014-2023

eTable 1. Median Number of Reviews by Facility Type

eFigure 2. Difference in Mean Platform Health Facility Positive Rating Pre/Post March 2020

eFigure 3. Interrupted Time Series (ITS) Analysis of Positive Reviews Pre- and Post-COVID-19

eTable 2. Interrupted Time Series (ITS) Analysis of Positive Reviews Pre- and Post-COVID-19: Coefficients and 95% Confidence Intervals for Changes in Review Positivity by Facility Type, Racial/Ethnic Composition, and Urban/Rural Settings

eTable 3. Multivariate Relationship Between Platform Health Facility Rating Positivity and Facility Characteristics and Time

eTable 4. Topic Coherence and Uniqueness Scores by Model

eTable 5. Themes Associated With Platform Health Facility Reviews

eFigure 4. Change in Theme Prevalence Over Time Among Positive Reviews

This supplementary material has been provided by the authors to give readers additional information about their work.

© 2024 Sehgal NKR et al. *JAMA Network Open.*

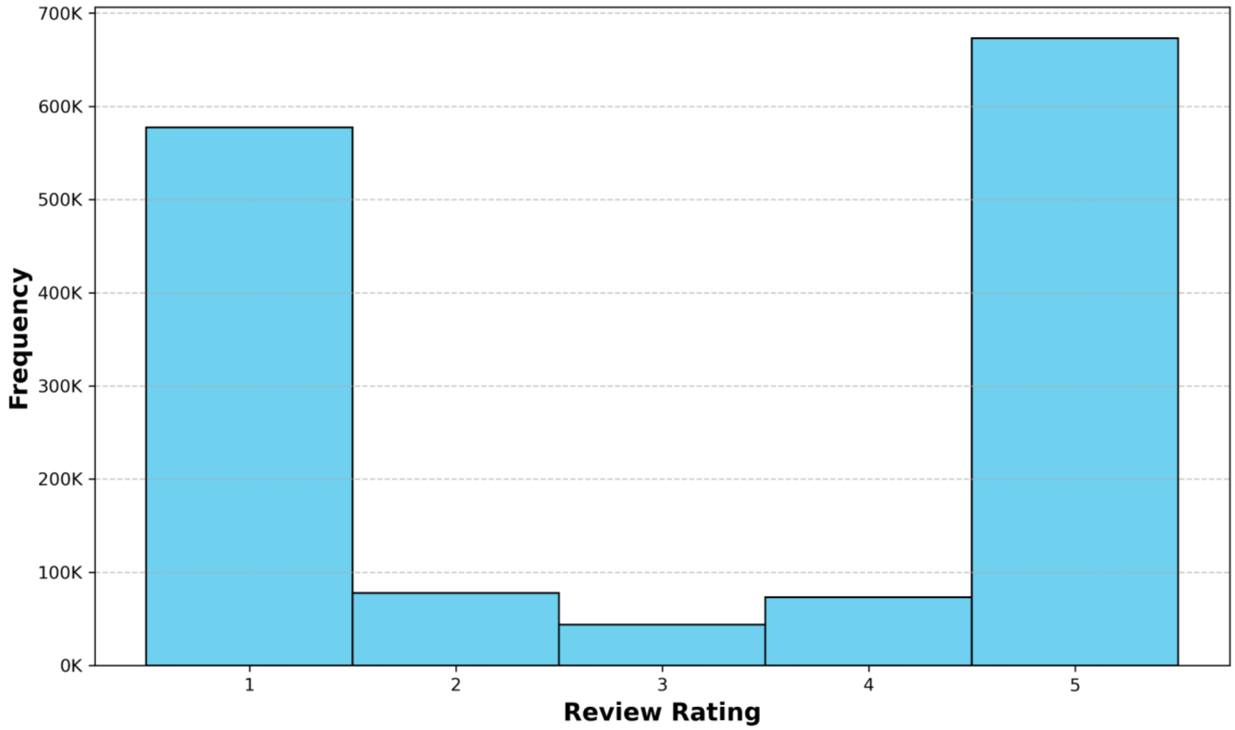

eFigure 1. Distribution of Platform Health Facility Ratings, 2014-2023

© 2024 Sehgal NKR et al. *JAMA Network Open.*

Facility Type	Median (IQR) Number of Reviews
Urgent Care	66 (116)
Medical Centers	55 (113)
Physical Therapy	21 (45)
Pharmacy	28 (48)
Hospitals	93 (180)
Physicians	12 (45)
Counseling & Mental Health	10 (26)
Rehabilitation Center	17 (33)
Emergency Rooms	110 (195)
Occupational Therapy	20 (39)
Home Health Care	11 (39)
Skilled Nursing	20 (33)
Ultrasound Imaging Centers	73 (177)
Lactation Services	39 (72)
Prenatal	41 (85)
Speech Therapists	13 (24)
Diagnostic Services	45 (115)
Reproductive Health Services	65 (127)
Nurse Practitioner	18 (56)
Dialysis Clinics	2 (5)

eTable 1. Median Number of Reviews by Facility Type

© 2024 Sehgal NKR et al. *JAMA Network Open.*

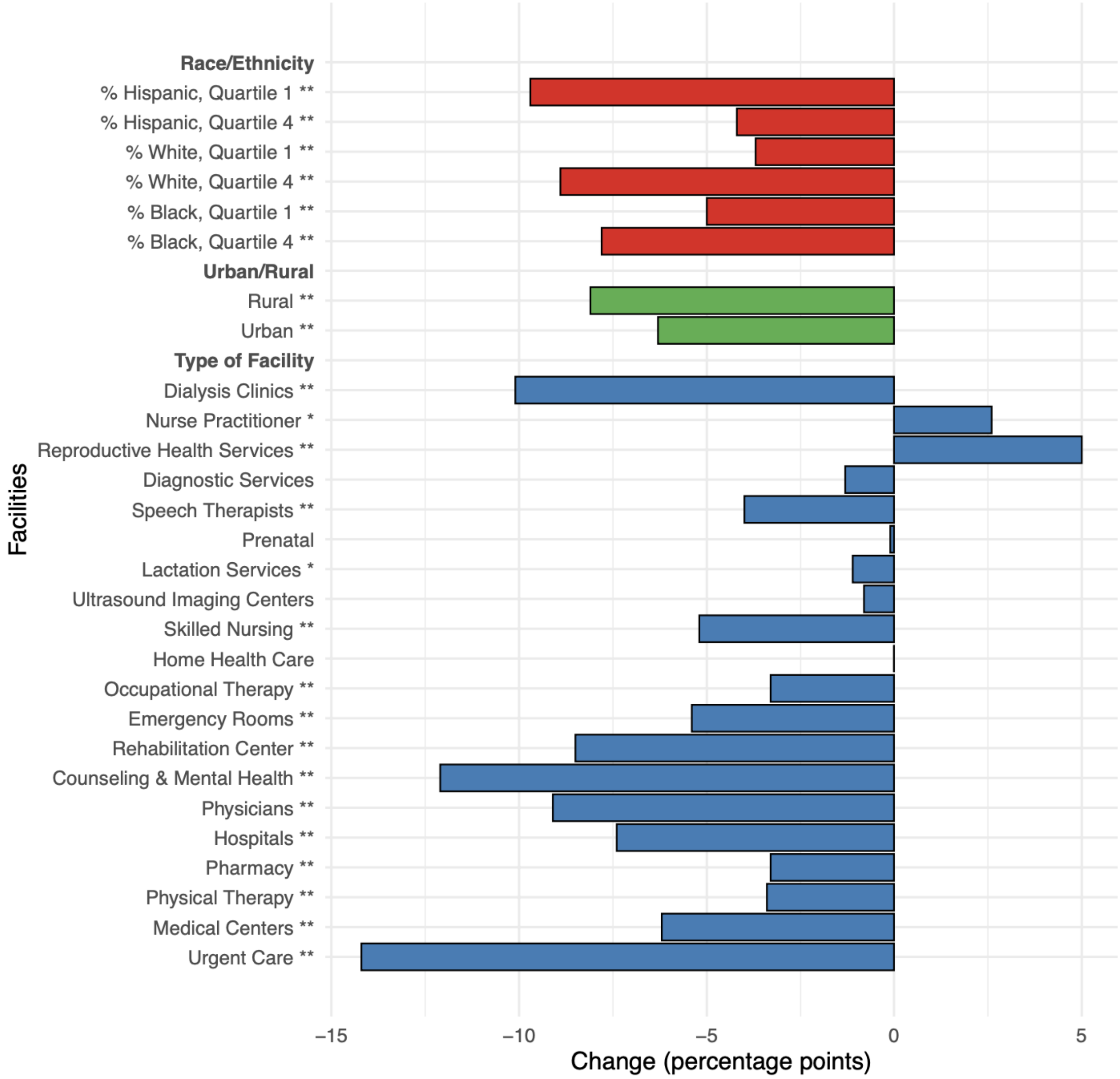

eFigure 2. Difference in Mean Platform Health Facility Positive Rating Pre/Post March 2020

Bars marked with * indicate P < 0.05, and ** indicate P < 0.001

© 2024 Sehgal NKR et al. *JAMA Network Open.*

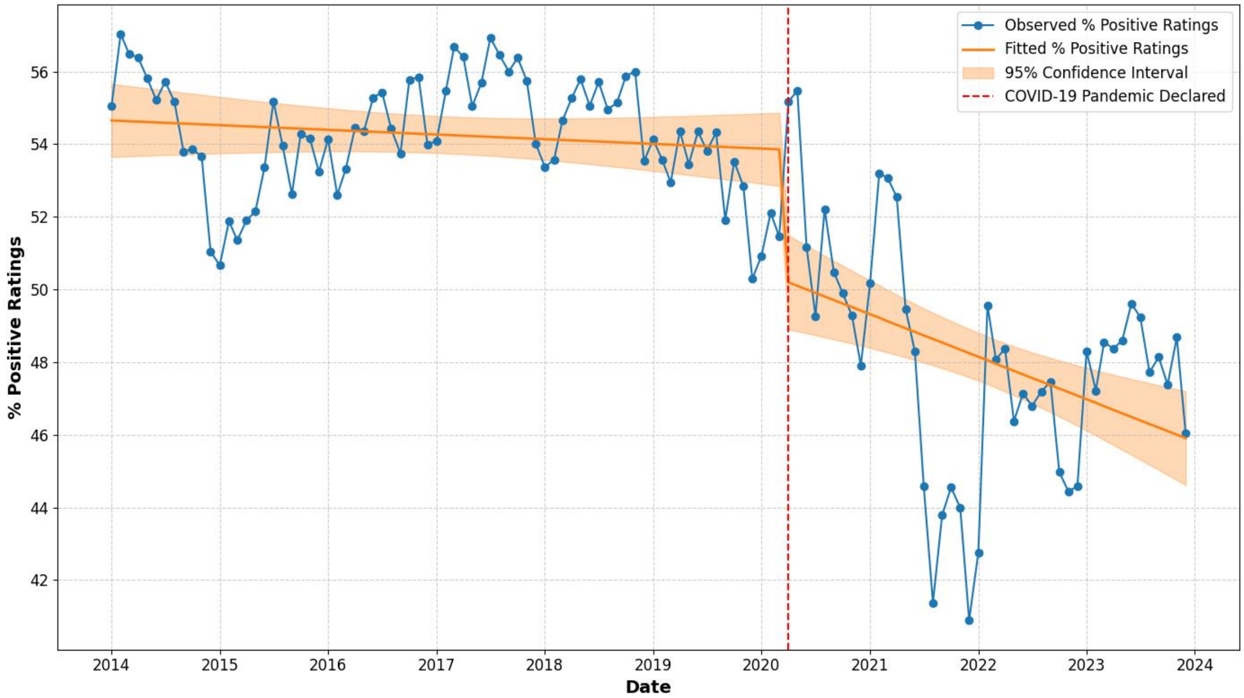

eFigure 3. Interrupted Time Series (ITS) Analysis of Positive Reviews Pre- and Post-COVID-19

© 2024 Sehgal NKR et al. *JAMA Network Open.*

Group	Post-Covid Level Change (95% CI)	Post-Covid Trend Change (95% CI)
Overall	0.030 (-0.022, 0.081)	-0.001** (-0.001, -0.000)
**Facility Type**		
Urgent Care	-0.144* (-0.264, -0.023)	0.000 (-0.004, 0.004)
Medical Centers	-0.013 (-0.085, 0.058)	-0.001 (-0.003, 0.002)
Physical Therapy	0.105*** (0.060, 0.149)	-0.004*** (-0.006, -0.003)
Pharmacy	0.115* (0.013, 0.216)	-0.004* (-0.008, -0.001)
Hospitals	-0.029 (-0.091, 0.033)	-0.000 (-0.002, 0.002)
Physicians	-0.047 (-0.138, 0.044)	-0.001 (-0.004, 0.002)
Counseling & Mental Health	0.012 (-0.049, 0.073)	-0.002 (-0.004, 0.000)
Rehabilitation Center	-0.015 (-0.082, 0.053)	-0.001 (-0.004, 0.001)
Emergency Rooms	-0.074 (-0.188, 0.039)	0.000 (-0.004, 0.004)
Occupational Therapy	0.020 (-0.077, 0.117)	-0.003 (-0.006, 0.000)
Home Health Care	0.276*** (0.163, 0.389)	-0.011*** (-0.015, -0.007)
Skilled Nursing	-0.132 (-0.274, 0.010)	0.002 (-0.003, 0.007)
Ultrasound Imaging Centers	0.256*** (0.164, 0.348)	-0.009*** (-0.012, -0.006)
Lactation Services	0.089** (0.037, 0.141)	-0.003** (-0.005, -0.001)
Prenatal	-0.011 (-0.076, 0.054)	0.000 (-0.002, 0.002)
Speech Therapists	0.081 (-0.019, 0.180)	-0.004* (-0.007, -0.000)
Diagnostic Services	0.117 (-0.026, 0.260)	-0.005* (-0.010, -0.000)
Reproductive Health Services	-0.217** (-0.352, -0.081)	0.007** (0.002, 0.012)
Nurse Practitioner	0.336*** (0.160, 0.511)	-0.012*** (-0.018, -0.006)
Dialysis Clinics	-0.002 (-0.329, 0.325)	0.000 (-0.011, 0.011)
**Urban/Rural**		

© 2024 Sehgal NKR et al. *JAMA Network Open.*

Rural	-0.096 (-0.205, 0.014)	0.002 (-0.002, 0.005)
Urban	0.042 (-0.032, 0.117)	-0.003* (-0.006, -0.001)
**% Black**		
Quartile 4	0.009 (-0.066, 0.084)	-0.002 (-0.005, 0.000)
Quartile 1	0.065 (-0.009, 0.139)	-0.003** (-0.006, -0.001)
**% White**		
Quartile 4	0.008 (-0.074, 0.089)	-0.002 (-0.005, 0.000)
Quartile 1	0.043 (-0.024, 0.111)	-0.003* (-0.005, -0.000)
**% Hispanic**		
Quartile 4	0.040 (-0.037, 0.117)	-0.003* (-0.006, -0.000)
Quartile 1	-0.034 (-0.119, 0.050)	-0.001 (-0.004, 0.002)

eTable 2. Interrupted Time Series (ITS) Analysis of Positive Reviews Pre- and Post-COVID-19: Coefficients and 95% Confidence Intervals for Changes in Review Positivity by Facility Type, Racial/Ethnic Composition, and Urban/Rural Settings.

© 2024 Sehgal NKR et al. *JAMA Network Open.*

Characteristic	Odds Ratio (95% confidence interval)
Post-COVID	0.73 (0.66, 0.81)
**Facility Type**	
Urgent Care	0.82 (0.81, 0.83)
Medical Centers	0.55 (0.55, 0.56)
Physical Therapy	2.75 (2.71, 2.79)
Pharmacy	0.44 (0.43, 0.44)
Hospitals	0.44 (0.43, 0.44)
Physicians	0.65 (0.64, 0.66)
Counseling & Mental Health	1.15 (1.13, 1.17)
Rehabilitation Center	0.81 (0.8, 0.83)
Emergency Rooms	1.05 (1.03, 1.07)
Occupational Therapy	0.55 (0.53, 0.56)
Home Health Care	0.86 (0.84, 0.88)
Skilled Nursing	0.57 (0.55, 0.58)
Ultrasound Imaging Centers	1.87 (1.81, 1.92)
Lactation Services	6.52 (6.21, 6.84)
Prenatal	2.54 (2.45, 2.64)
Speech Therapists	1.79 (1.72, 1.85)
Diagnostic Services	0.84 (0.82, 0.87)
Reproductive Health Services	1.96 (1.89, 2.03)
Nurse Practitioner	1.87 (1.77, 1.97)
Dialysis Clinics	0.73 (0.66, 0.81)
**Urban/Rural**	
Rural	0.95 (0.93, 0.97)
Urban	--
**% Black**	
Quartile 4	0.58 (0.57, 0.59)

© 2024 Sehgal NKR et al. *JAMA Network Open.*

Quartile 2-3	0.83 (0.82, 0.84)
Quartile 1	--
**% White**	
Quartile 4	0.75 (0.74, 0.76)
Quartile 2-3	0.93 (0.93, 0.94)
Quartile 1	--
**% Hispanic**	
Quartile 4	1.05 (1.03, 1.06)
Quartile 2-3	1.15 (1.13, 1.16)
Quartile 1	--

eTable 3. Multivariate Relationship between Platform Health Facility Rating Positivity and Facility Characteristics and Time. Note: Each facility type is treated as a separate binary indicator variable as a facility can belong to multiple categories (e.g., Hospital and Emergency Room).

© 2024 Sehgal NKR et al. *JAMA Network Open.*

Model	Number of Topics	Uniqueness	C_{UMass}	C_v	C_{UCI}	C_{NPMI}
LDA	200	0.63	-3.96	0.62	1.10	0.11
LDA	500	0.52	-5.09	0.58	0.88	0.10
LDA	1000	0.45	-6.06	0.56	0.69	0.09
BERTopic	2000	0.84	-5.61	0.46	-0.08	0.05

eTable 4. Topic Coherence and Uniqueness Scores by Model

© 2024 Sehgal NKR et al. *JAMA Network Open.*

Theme	Top 10 Words
**Pharmacy**	
Pharmacy Services	clinic, pharmacy, years, ready, prescription, line, lady, pick, prescriptions, name
Pharmacy and Prescription Services	pharmacy, cvs, service, customer, store, prescription, pharmacist, prescriptions, call, ready
Medication and Prescription Management	medication, facility, prescription, nursing, meds, body, refill, exercises, call, rehab
Pharmacy Communication	stars, give, years, call, phone, star, pharmacy, minutes, cvs, hold
**Services**	
Pediatric Care	visit, kids, child, first, children, questions, store, cvs, treatment, explained
Therapy	life, our, us, thank, therapy, baby, pharmacy, health, physical, therapist
Communication and Insurance Handling	call, phone, called, insurance, review, office , medical, questions, answer, calls
Facility Cleanliness and Wait Time	nice, store, room, it's, cvs, clean, really, wait, front, always
Family Medicine and Insurance	family, therapy, facility, physical, him, mom, room, office , er, insurance
Family Member Treatment	son, him, surgery, our, us, mom, number, old, name, information
Emergency Room Services	er, room, waiting, emergency, hours, blood, nurses, nurse, him, test
Family Health Care	our, us, him, daughter, husband, baby, mom, family, call, mother
Physical Therapy and Injury Rehabilitation	therapy, physical, pt, therapist, surgery, knee, exercises, him, therapists, injury
**Communication**	
Customer Service and Staff Behavior	front, desk, rude, service, customer, him, ever, worst, unprofessional, room
Staff Friendliness and Professionalism	recommend, urgent, front, nice, always, highly, helpful, desk, friendly, thank
**Logistics**	
Wait Time	minutes, wait, room, waiting, hour, hours, waited, er, than, 30

© 2024 Sehgal NKR et al. *JAMA Network Open.*

Billing and Insurance Charges	insurance, pay, bill, money, test , paid, billing, charge, charged, covid
Insurance and Billing Issues	insurance, billing, office , times, i've, bill, always, company, call, both
Test Results and Wait Time	him, i've, wait, ever, it's, test, results, office, else, waiting
Scheduling and Parking	store, appointments, parking, scheduled, appt, schedule, surgery, results, cvs, minutes

eTable 5. Themes Associated with Platform Health Facility Reviews

© 2024 Sehgal NKR et al. *JAMA Network Open.*

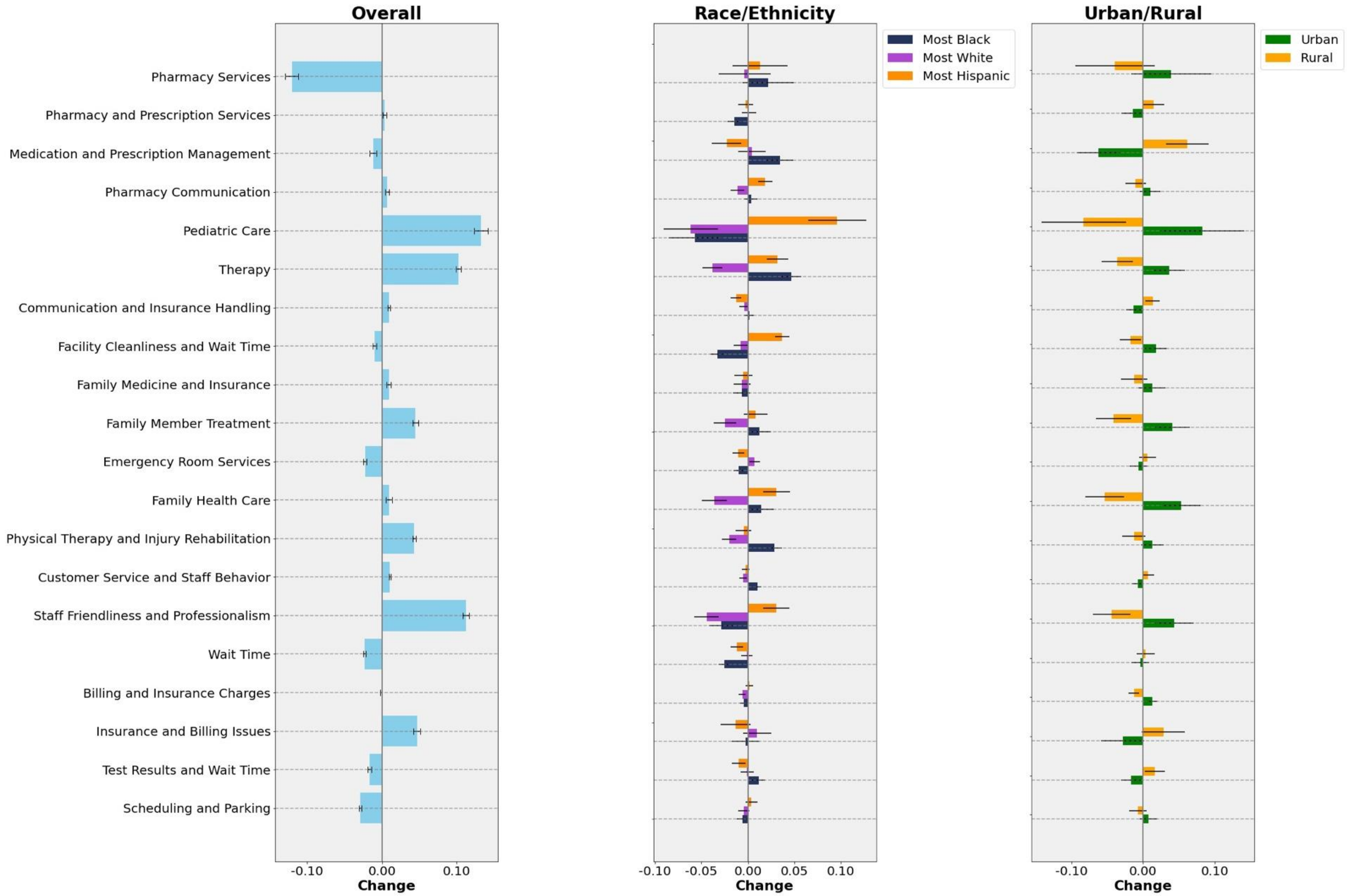

eFigure 4. Change in Theme Prevalence Over Time Among Positive Reviews. Panel A) All Positive Reviews. Panel B) Race/Ethnicity Quartile 4 vs Quartile 1. Panel C) Urban vs Rural

© 2024 Sehgal NKR et al. *JAMA Network Open.*
