# Supplementary material for: Disparities by Race and Urbanicity in Online Health Care Facility Reviews
Source: JAMA Netw Open. 2024 Nov 22;7(11):e2446890. doi: 10.1001/jamanetworkopen.2024.46890 (PMC11584935; doi:10.1001/jamanetworkopen.2024.46890)
Supplement: Supplement 2. — Data Sharing Statement [file jamanetwopen-e2446890-s002.pdf]

Data Sharing Statement

Sehgal. Disparities by Race and Urbanicity in Online Health Care Facility Reviews. *JAMA Netw Open*. Published November 22, 2024. doi:10.1001/jamanetworkopen.2024.46890

Data
Data available: No
